# Developmental Reorganization of the Cognitive Network in Pediatric Epilepsy

**DOI:** 10.1371/journal.pone.0141186

**Published:** 2015-10-27

**Authors:** Camille Garcia-Ramos, Jack J. Lin, Vivek Prabhakaran, Bruce P. Hermann

**Affiliations:** 1 Department of Medical Physics, University of Wisconsin-Madison, Madison WI, United States of America; 2 Department of Neurology, University of California, Irvine, Irvine CA, United States of America; 3 Department of Radiology, University of Wisconsin-Madison, Madison WI, United States of America; 4 Department of Neurology, University of Wisconsin-Madison, Madison WI, United States of America; Chinese Academy of Sciences, CHINA

## Abstract

Traditional approaches to understanding cognition in children with epilepsy (CWE) involve cross-sectional or prospective examination of diverse test measures, an approach that does not inform the interrelationship between different abilities or how interrelationships evolve prospectively. Here we utilize graph theory techniques to interrogate the development of cognitive landmarks in CWE and healthy controls (HC) using the two-year percentage change across 20 tests. Additionally, we characterize the development of cognition using traditional analyses, showing that CWE perform worse at baseline, develop in parallel with HC, statically maintaining cognitive differences two years later. Graph analyses, however, showed CWE to exhibit both lower integration and segregation in development of their cognitive networks compared to HC. In conclusion, graph analyses of neuropsychological data capture a dynamic and changing complexity in the interrelationships among diverse cognitive skills, maturation of the cognitive network over time, and the nature of differences between normally developing children and CWE.

## Introduction

It is widely appreciated that neuropsychological status can be adversely affected in the childhood epilepsies, even among the so-called benign idiopathic epilepsies where intelligence is typically average but with abnormalities in specific areas of cognitive ability including language, memory, executive function or processing speed [1–2]. Prospective investigations tracking children from the time of onset and diagnosis indicate that cognitive differences are present at or near the time of diagnosis with these differences typically maintained over time, without evidence of progressive decline or significant improvement out to five years later [3–5].

These cognitive patterns have been uncovered and characterized by analysis of individual test scores (e.g., intelligence), combinations of test scores (e.g., intelligence, memory, executive function), or factor scores obtained at baseline and prospectively in some studies. While cognitive differences between participants with epilepsy and normally developing children can be identified and tracked over time, unclear however is how these diverse cognitive abilities and domains interact with one another in the epilepsy and control participants, if the interrelationships are different, and if so, in what ways. Especially unclear is the trajectory of these cognitive interrelationships and networks in the context of maturational brain changes, as increasing chronological age is of course associated with the development, specialization and harmonization of discrete cognitive skills (e.g., the emergence of improved executive functioning in adolescence) [6].

A new approach to examine these interrelationships among diverse cognitive abilities is to conceptualize cognition as a *network* in which cognitive skills are arranged in an optimal balance between integration and segregation [7]. Such an approach, using graph theory techniques [8–9], is grounded in empiric experiments that demonstrated cognition is not simply the summation of discrete abilities but is a dynamic network in which training in one cognitive domain (e.g. working memory and executive function) augments skills in a different domain (e.g. fluid intelligence and language skills) [10–11]. Further, the interrelationship between cognitive abilities is impacted by aging, as diverse abilities becomes more inter-correlated, or dedifferentiated in old age, presumably reflecting a neurological process that impairs global rather than domain specific cognition [12].

Graph theory has been utilized in wide variety of imaging research to examine brain networks in epilepsy [13–15], as well as to examine relationships between brain networks and cognition [16], but rarely has cognition been the sole focus of examination. In the first characterization of cognition in epilepsy using graph theory techniques, we showed that the cognitive network was abnormal in children with newly diagnosed idiopathic epilepsies by virtue of less age appropriate segregation of cognitive skills and fragmentation of other ability areas compared to normally developing children [17]. While our cross-sectional study validated this approach and demonstrated baseline differences in epilepsy and healthy controls, this study examines their prospective cognitive development over a 2-year interval.

In so doing, we hypothesize that patterns of cognitive development in the epilepsy participants will reveal network alterations that facilitate developmental interactions among cognitive domains (hubs) to maintain average intelligence overtime; nevertheless, such a configuration will be more disorganized compared to normally developing controls. The measures to be calculated to explore these developmental issues include graph clustering coefficient, harmonic mean, modularity, and betweenness centrality; the latter making it possible to identify network hubs or the relative importance of nodes in the cognitive developmental network in each group. Finally, we hypothesize that our conceptualization of prospective cognitive network changes using graph theory will provide distinct insight into the dynamic coordination of different abilities over time that are not afforded by traditional analyses of neuropsychology data.

## Methods

### Participants

Research participants consisted of 178 youth aged 8–18, including 104 with new and recent-onset epilepsy and 74 healthy first-degree cousin controls. All participants had completed two waves of neuropsychological assessment including baseline and 2-year follow-up evaluations. At baseline, all participants attended regular schools. Children with epilepsy were recruited from pediatric neurology clinics at three midwestern medical centers (University of Wisconsin-Madison, Marshfield Clinic, Dean Clinic) and met the following inclusion criteria: (i) diagnosis of epilepsy within the past 12 months; (ii) no other developmental disabilities (e.g. intellectual impairment, autism); (iii) no other neurological disorder, and (iv) normal clinical MRI. All children entered the study with active epilepsy diagnosed by their treating pediatric neurologists and confirmed by medical record review of the research study pediatric neurologist. We did not exclude children on the basis of psychiatric comorbidities (including attention deficit hyperactive disorder) or learning disabilities. We did however exclude children with intellectual disabilities, autism, and /or other neurological disorders. Details regarding the subject selection process have been described in detail in previous publications [18]. In general, we tried to stay true to the concept of “epilepsy only” as defined broadly in literature [3], characterized by normal neurological exams, intelligence, and attendance at regular schools. Each child’s epilepsy syndrome (Idiopathic Generalized or Localization Related Epilepsy) was defined in a research consensus meeting by the research pediatric neurologist who reviewed all available clinical data (e.g., seizure description and phenomenology, EEG, clinical imaging, neurodevelopmental history) while blinded to all research cognitive, behavioral, and neuroimaging data.

First-degree cousins were used as controls and exclusion criteria were as follows: (i) history of any initial precipitating insult (e.g. simple or complex febrile seizures, cerebral infections, perinatal stroke); (ii) any seizure or seizure-like episode; (iii) diagnosed neurological disease; (iv) loss of consciousness greater than 5 min; (v) other family history of a first-degree related with epilepsy or febrile convulsions. For the current analysis we also excluded control participants with education services (n = 9). We used cousin controls rather than siblings or other potential control groups for the following reasons: (i) first-degree cousins are more genetically distant from the participants with epilepsy and thus less pre-disposed than siblings to shared genetic factors that may contribute to anomalies in brain structure and cognition; (ii) a greater number of first-degree cousins are available than siblings in the target age range and (iii) the family link was anticipated to facilitate participant recruitment and especially retention over time (which is our intent) compared to more general control populations (e.g. unrelated school mates). A summary of demographic and clinical characteristics of participants can be found in Table 1.

**Table 1:** Demographic and Clinical Characteristics.

	Controls (*n* = 74)	Epilepsy (*n* = 104)
Age (mean ± SD)	12.5 ± 3.1	12.2 ± 3.2
Gender (F/M)	35/39	53.51
Grade level (mean ± SD)	6.2 ± 2.9	6.1 ± 3.2
IQ (mean ± SD)*	108.7 ± 11.1	101.0 ± 13.5
Parental education (mean ± SD)	4.7 ± 1.5	4.4 ± 1.7
AED (yes/no)	-	88/16
Syndrome duration (months: mean ± SD)	-	8.5 ± 3.5
Epilepsy syndrome	-	ILRE = 55IGE = 49

*Significantly different between groups; ILRE = idiopathic localization-related epilepsy; IGE = idiopathic generalized epilepsy.

### Neuropsychological Assessment

To comprehensively evaluate cognition, a battery of neuropsychological tests were administered to all youth that yielded 20 test measures reflecting performance across the domains of intelligence, academic achievement, language, memory, executive function, and cognitive/psychomotor speed. These test scores were standardized according to age norms in both control and epilepsy groups in order to proceed with graph analyses. The details for all tests and targeted cognitive domains are contained in Table 2.

**Table 2:** Cognitive tests for graph calculation.

	Abbreviation	Name	Cognitive ability	Cognitive domain
1	IQVOCS	WASI Vocabulary	Verbal intelligence	Intelligence
2	IQBDS	WASI Block Design	Nonverbal intelligence	Intelligence
3	IQSIMS	WASI Similarities	Verbal intelligence	Intelligence
4	IQMRS	WASI Matrix Reasoning	Nonverbal intelligence	Intelligence
5	READSTN	WRAT-IV Reading	Word recognition	Academic achievement
6	SPELSTN	WRAT-IV Spelling	Spelling	Academic achievement
7	ARITSTN	WRAT-IV Arithmetic	Arithmetic calculation	Academic achievement
8	PPVTSTN	Peabody Picture Vocabulary Test	Language (word recognition)	Language
9	EVTSTN	Expressive Vocabulary Test	Language (word naming)	Language
10	LETFLUS	D-KEFS Letter Fluency	Language (phonemic fluency)	Language
11	BNTTOT	Boston Naming Test	Language (naming)	Language
12	WLLSS	Children’s Memory Scale-III	Immediate Verbal Memory	Memory
13	WLDSS	Children’s Memory Scale-III	Delayed Verbal Memory	Memory
14	CATFLUS	D-KEFS Category Fluency	Executive function (semantic fluency)	Executive
15	CATSWS	D-KEFS Category Switching	Executive function (category switching)	Executive
16	INHSS	D-KEFS Inhibition	Executive function (response inhibition)	Executive
17	CORSORS	D-KEFS Card Sorting Test	Executive function (problem solving)	Executive
18	IQDSYMS	WISC-IV Digit Symbol	Speed	Speed
19	COLSS	D-KEFS Color-Word	Speed	Speed
20	WORDSS	D-KEFS Word Reading	Speed	Speed

In addition to graph analyses we characterized cognitive development in a traditional fashion by examining change over time across individual test measures assessing the domains of intelligence (Composite IQ), executive function (D-KEFS Card Sorting Test), language (Expressive Vocabulary Test [EVT]), and processing speed (WISC-IV Digit Symbol) between control subjects and children with epilepsy. This was done using a 2 (Groups: Controls and Epilepsy) x 2 (Time: Baseline and Follow-up) mixed repeated measures ANCOVA with age and sex as covariates. These analyses stand as a conventional analysis of neuropsychological data to which the results of the analyses using graph theory could be compared.

### Network Analysis

In this study, we focused on the prospective changes in cognitive network between the two groups rather than comparing performances of the groups at two time points because scores between groups might differ at baseline evaluation. Therefore we normalized to the baseline performance when examining the difference between test scores at both time points (baseline and two-year follow-up) and the result was multiplied by 100 in order to obtain the percentage change for each group according to $\frac{{TP}_{2}-{TP}_{1}}{{TP}_{1}}x100$, were TP_1_ and TP_2_ are the baseline and follow-up scores, respectively.

Subsequently, a graph or network of correlation coefficients based on the covariance between the percentage changes between tests was constructed for each group rendering symmetric (undirected) weighted adjacency matrices of 20 nodes, *N* (tests) and 190 edges (connections between them; (*N* × (*N* − 1))/2). Afterwards, global measures were acquired at different graph densities while local measures were calculated at a density level that fully connected the networks in all groups (epilepsy and control).

One method commonly used to perform graph thresholding is *proportional thresholding*, which ensures that only X% of the strongest (highest weighted) links form the graph. For example, a proportional threshold of 5% in a matrix of 20 nodes means that only the strongest 10 links would be included in the graph analysis (5% of the 190 total connections in the graph). Unfortunately at lower thresholds graphs might not be fully connected, making group comparisons inaccurate. In order to ensure a fully connected graph for the analysis there is another thresholding method called the *Minimum Spanning Tree* (MST), which is a subgraph of the network that connects all nodes in the graph while minimizing the connection distance (using the strongest weights) between them without forming cycles [19]. However, MST ignores links that form loops, which are necessary for the proper investigation of networks. To harvest the advantage of MST and proportional thresholding methods while avoiding their shortcomings, we used a novel approach to combine these two methods in three steps. First, we calculated the graph minimum spanning tree (MST); second, we calculated the proportional threshold of interest from the graph without the MST; third, we added both results in order to have a fully connected graph at each level of graph density while ensuring the same number of edges for each group [20]. In this way the MST serves as the graph skeleton that ensures that no node is disconnected, and each added proportional threshold helps in the investigation of the interactions of interest. For the remainder of this manuscript, each graph threshold represents a combination of MST and proportional thresholding, indexed by the density level. For example, a density level of 15% would be the MST plus a proportional threshold of 15%. Global metrics were calculated over a range of graph connectivity densities from 5 to 40%, and local measures were calculated at a density level of 20% given that this level was the closest to the average of full graph connectedness in both groups. The Force Atlas algorithm of the open source software *Gephi* (http://gephi.github.io/) was used for the 2D visualization of community structure on each group (attraction strength = 10, repulsion strength = 1000, gravity = 30).

### Graph Theory Measures

Graph theoretical measures were obtained using the Matlab based Brain Connectivity Toolbox (BCT, http://www.brain-connectivity-toolbox.net/). Weighted-undirected adjacency matrices were created to obtain different graph theory metrics. In order to discern statistically significant group differences, each group matrix was resampled by replacement (i.e. bootstrapped) a total of 1000 times. Graph theory measures were obtained from each resampled matrix at the same threshold and averages were used for evaluations. The graph theory measures of interest in this investigation are described below.

#### Harmonic mean

Harmonic mean, *H*_*m*_ is a measure of integration in a network. It is defined as the inverse of the global efficiency, *E* which is based on the shortest paths or direct connections between nodes, *N* [21]: Hm=1E=N(N−1)∑i,j∈G1di,j, where *d*_*i*,*j*_ is the distance from node *i* to node *j*. Low *H*_*m*_ reflects higher graph integration reflecting a more efficient network.

#### Clustering coefficient

The graph clustering coefficient is a measure that reflects how clustered a network is. It is calculated as the ratio of “triangles” (closed connection between three nodes) to “triplets” (connections between three nodes) in the network [22–23]: $T=\frac{3\;\times \;\#\;of\;triangles\;in\;G}{\#\;of\;connected\;triples\;of\;vertices\;in\;G}$. Therefore, it lies between 0 and 1, with low values (close to 0) indicating low clustering and values close to 1 indicating a highly clustered network.

#### Modularity index and community structure

The modularity or community structure of a network is the subdivision of a network into segregated communities that contribute to the same processes. Unlike the other measures, modular/community structure is statistically estimated instead of computed exactly [24]. Since the number of communities could be variable with different iterations, community structure was calculated 100 times for each group matrix and the number with the highest likelihood was chosen. The modularity index is a measure of the goodness of the subdivision of the graph into communities; the higher its value, the stronger the modular structures in the graph [25]. Modular structure in a network is of great importance because it allows for different processes to take place, therefore representing specialization in the network.

#### Betweenness centrality

Betweenness centrality (BC) is a measure of the importance of a node, *i* for the communication of a network [25]. It is calculated based on the node degree and its closeness, which is just the inverse of the average distance from the other nodes; defined as $b_{i}\;=\;\sum_{j,k\in N,j\neq k}\frac{n_{jk}\left(i\right)}{n_{jk}}$, where *n*_*jk*_ is the number of shortest paths connecting nodes *j* and *k*, while *n*_*jk*_ *(i)* is the number of shortest paths connecting *j* and *k* and passing through node *i*. Nodes with high betweenness centrality facilitate global integrative processes given that they serve as “highways” to facilitate “traffic” flow of the network [26].

These metrics were computed for the adjacency matrices of each group. The identical subjects were represented in baseline and follow-up assessments, thus rendering a clear picture of prospective change over time. As such, the combination of information from these metrics would inform about the nature of the development of the cognitive networks of both groups in a two-year period. Statistical testing for the developmental change between healthy controls and children with epilepsy was performed, correcting for multiple comparisons using Bonferroni correction.

## Results

Organizational conformations of cognitive network development were investigated in children with epilepsy at/near the time of diagnosis and in healthy controls. There were no significant differences between participants with epilepsy and healthy controls in age, gender, grade level, and socioeconomic status (*p* > 0.05).

### Neuropsychological Testing

**Fig 1:**
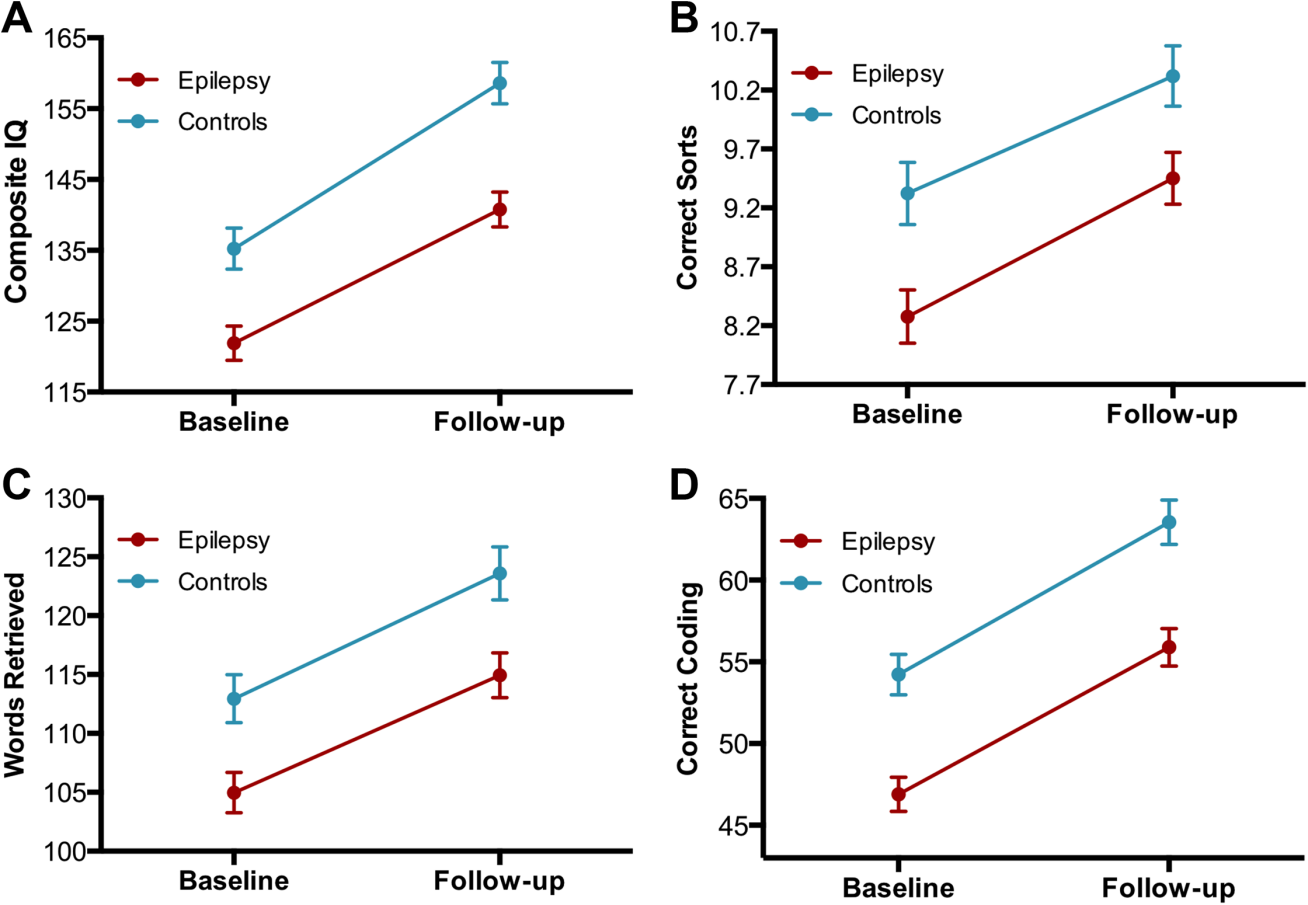
Prospective change in cognitive tests. Prospective change between subjects with epilepsy (red) and control participants (blue) in (A) composite IQ, (B) correct sorts (D-KEFS Card Sorting Test), (C) expressive vocabulary (EVT), and (D) processing speed (WISC-IV Digit Symbol). All tests showed main effects for Group in which, evidently, subjects with epilepsy were lower than HC. Analyses were corrected for age and gender.

Fig 1 presents the change from baseline to follow-up between controls and participants with epilepsy across individual tests representing the domains of intelligence, executive function, language, and processing speed using repeated measures ANCOVA. All tests showed main effects for group in which control subjects always performed better than the epilepsy participants over time (Composite IQ [27]: *F*(1,174) = 18.5, *p* < 0.001; correct sorts (D-KEFS Card Sorting Test) [28]: *F*(1,174) = 10.4, *p* = 0.002; expressive vocabulary (EVT) [29]: *F*(1,174) = 9.8, *p* = 0.002; processing speed (WISC-IV Digit Symbol) [30]: *F*(1,174) = 23.4, *p* < 0.001). Main effects for time (regardless of group) revealed significant improvement for the tests of Composite IQ [*F*(1,174) = 103.6, *p* < 0.001], expressive vocabulary [*F*(1,174) = 32.4, *p* < 0.001], and processing speed [*F*(1,174) = 34.1, *p* < 0.001]. None of the tests revealed significant Group x Time interactions. As depicted in Fig 1, the “parallel lines” across measures depict significant group differences at baseline, cognitive development or growth over the interval, the baseline cognitive differences maintained two years later across representative neuropsychological test measures.

### Graph Theory Metrics

**Fig 2:**
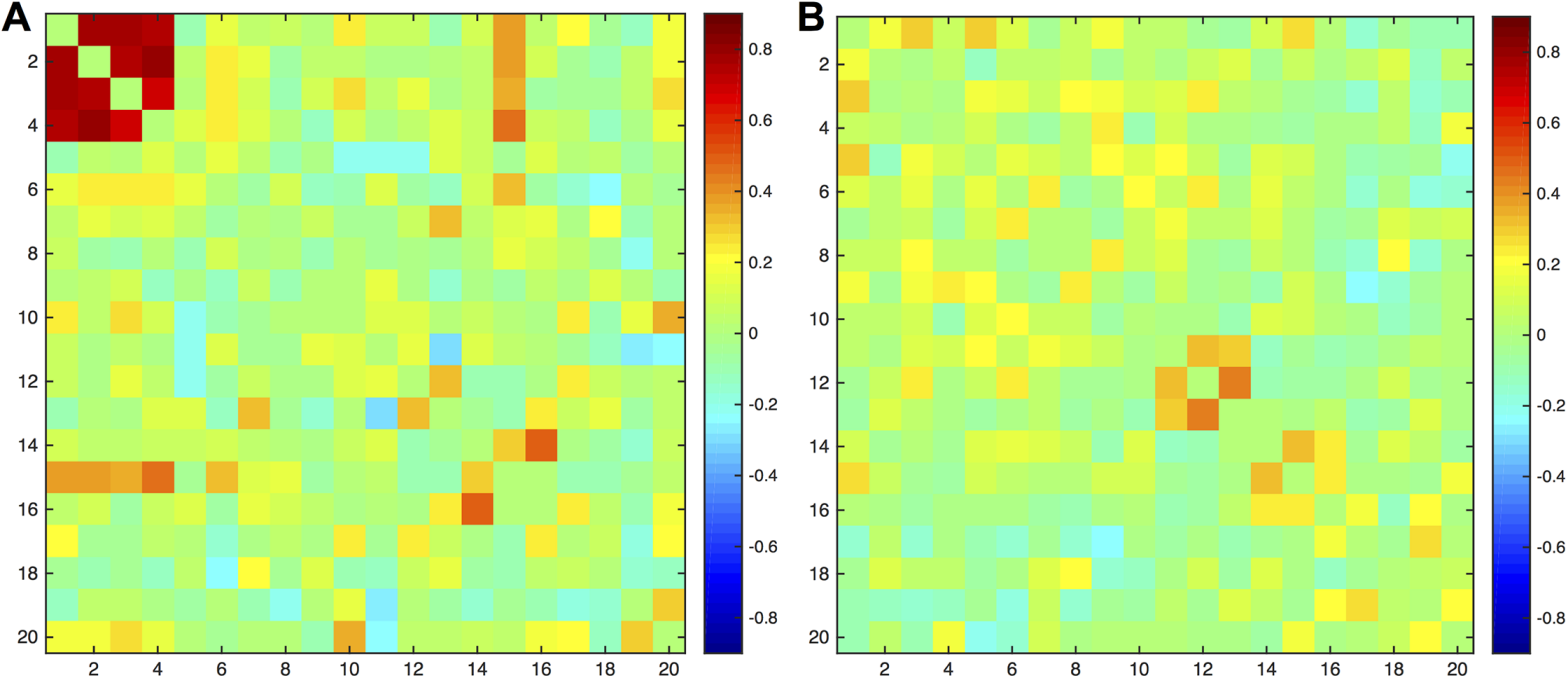
Adjacency matrices. Adjacency matrices for the correlation of the percentage change between 20 tests in (A) healthy controls and (B) subjects with epilepsy. Strong correlations are evident in the group of controls between the four IQ measures. The order of the nodes is the same as presented in Table 2.

Adjacency matrices for the correlation of the percentage change over two years (from baseline to follow-up) between tests in each group can be found in Fig 2, where differences in cognitive development between the two groups can be visually appreciated. The control group presented strong positive developmental correlations between the four IQ measures (first four tests) that were absent in subjects with epilepsy. Furthermore, the test of category switching (CATSWS) presented high correlations with those four IQ tests and also showed strong correlations with the test of inhibition (INHSS) in healthy controls, all of which were absent in the participants with epilepsy. In subjects with epilepsy there were strong positive correlations between naming (BNTTOT) and immediate memory (WLLSS) and between naming and delayed memory recall (WLDSS), the latter presenting instead negative correlations in controls.

#### Global integration and segregation

**Fig 3:**
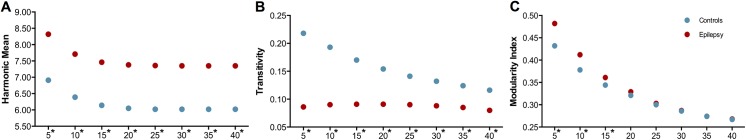
Global graph theory measures. (A) Harmonic mean, (B) graph clustering, (C) modularity index for the cognitive development in healthy controls (blue) and subjects with epilepsy (red). *Significantly different between groups (corrected for multiple comparisons).

The participants with epilepsy presented a significantly higher harmonic mean than healthy controls across the range of investigated graph densities (Fig 3A). Thus, participants with epilepsy presented lower developmental integration in their cognitive landmarks than healthy controls.

In terms of graph segregation, the epilepsy group showed significantly lower graph clustering than control participants across the range of graph densities (Fig 3B). Therefore, subjects with epilepsy presented lower global segregation than healthy controls in the development of their cognitive networks.

When examining modularity index, the epilepsy group showed higher modularity at 5–15% fixed density but both groups presented similar values above a density of 15% (Fig 3C).

#### Local segregation and centrality

**Fig 4:**
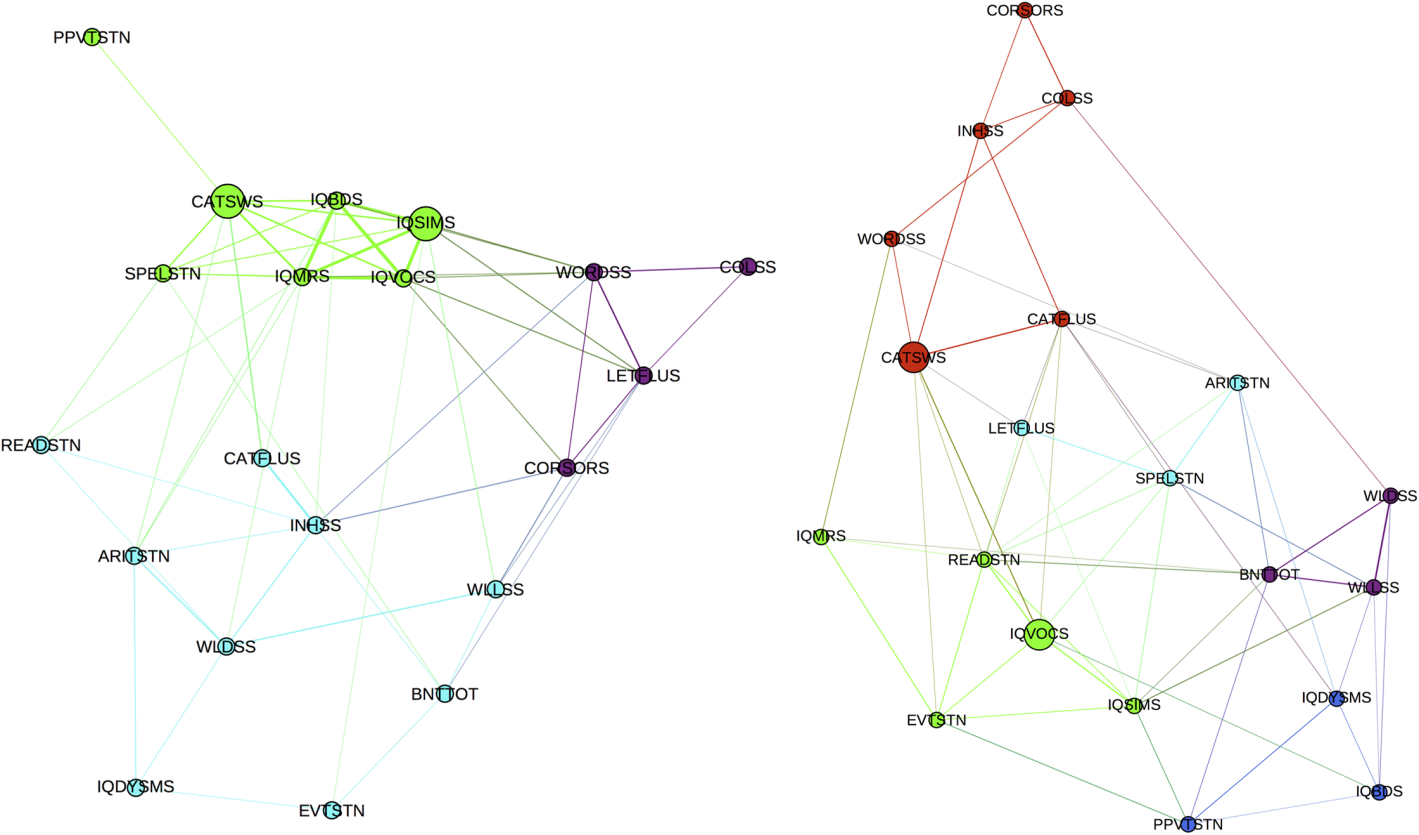
Community structure of cognitive networks. Community structure for the development of cognitive landmarks in healthy controls (left) and patients with epilepsy (right). Nodes with stronger and higher number of connections are spatially closer while those with weaker and lower number of connections are farther in space. Different colors represent different modules. Hubs are denoted with larger circles.

Community structure and betweenness centrality were investigated at a density of 20% for the development of cognitive landmarks of subjects with epilepsy and control participants (percentage change). As can be seen in Fig 4, healthy controls demonstrated cognitive development that showed a superior level of organization than the one observed in the participants with epilepsy. Control subjects presented three well-defined modules while subjects with epilepsy presented five modules with overlapping edges, which rendered a less defined developmental conformation.

In terms of hubs, or those tests that facilitate interactions between other tests, both groups presented the test of category switching (CATSWS) while also presenting a test of verbal intelligence as hub: controls showed verbal reasoning (IQSIMS) and participants with epilepsy showed vocabulary (IQVOCS). Therefore, verbal intelligence and executive function seem to be the cognitive abilities providing unification in the cognitive networks regardless of group (epilepsy or control). However, the hubs of verbal intelligence were not identical—verbal reasoning in the controls (i.e., using language in the service of thought and reasoning) while more concretely language based (i.e., defining words) in the participants with epilepsy.

In summary, subjects with epilepsy exhibit both lower global integration and segregation in the development of their cognitive networks compared to healthy controls. This network configuration seems to have affected the developmental organization of the cognitive networks in the participants with epilepsy. Although executive function and verbal intelligence served as hubs in both groups, control participants employed reasoning skills while children with epilepsy used more concrete verbal skills.

## Discussion

This application of graph theory techniques to prospective neuropsychological data in children with epilepsy and normally developing children provides a novel avenue to characterize and understand the impact of epilepsy and its treatment on cognitive networks and circuitry development, something heretofore not undertaken given the inherent limitations associated with traditional psychometric analyses of human cognitive data. This was shown in the following ways. First, the epilepsy participants showed significantly lower global integration and segregation in the development of their cognitive network than healthy controls. Second, even though both groups presented the same number and similar but not identical kinds of hubs, control subjects presented a more organized development compared to the children with epilepsy as visualized in their community structure. Third, high developmental correlations between measures of intelligence and executive function seemed to be a key component for the efficient and optimal development of cognitive network.

Comparison of the results using graph theory to traditional analyses of cognitive data provides a very divergent view of the effects of epilepsy on prospective cognitive development. Traditional developmental investigations examining tests from different cognitive domains have shown significant differences between children with epilepsy and healthy controls at baseline with comparable development over time, maintaining static group differences with further development [31–32]. As re-demonstrated here (Fig 1), both groups maintain the baseline differences that are again observed two years later with seemingly comparable developmental trajectories. This seems to suggest a fairly static “offset” at baseline, maintained at follow up, with otherwise comparable cognitive trajectories—a very consistent and arguably static pattern across multiple cognitive domains. When we explored cognitive development by examining the correlational differences between test score changes over a two-year time frame, a divergent picture of the actual conformational changes of the cognitive networks emerged in contrast to traditional psychometric analyses. Indeed, the global changes in cognitive development were quite different in children with epilepsy compared to control participants. Children with epilepsy presented lower global efficiency (higher harmonic mean) along with lower global clustering that led to a less robust modular organization (i.e. less distinct communities) of their cognitive development (Fig 4, right).

The global topology of the cognitive network of children with epilepsy revealed a shift toward a configuration in which both global efficiency and segregation were lower compared to controls [7]. Whereas this type of topology does not promote integration of information transfer given the sparse relationship between different tests from different domains (i.e. distant nodes), it is also suboptimal in processing of related tests (i.e. neighboring nodes) (Figs 2 and 4). Surprisingly the global modularity is not impaired in the epilepsy group compared to control. At lower threshold, the modularity was in fact higher in the epilepsy group compared to controls, but at higher threshold, no significant difference existed between the groups. Studies of brain development employing graph theory have demonstrated that global modularity is in general stable from childhood to adolescent [33–35]. We speculate that the conservation of modularity in the face of altered cognitive network configuration may underlie the overall maintenance of cognitive function of children with epilepsy in which intelligence remained at an average range despite deficits in other discrete cognitive domains.

Investigations of the local changes in cognitive development rendered similarities and differences between epilepsy and control subjects. The number of cognitive hubs was the same in both groups while also representing the similar cognitive domains of executive function and verbal intelligence. It is not surprising that a measure of executive function represented a hub in the network of healthy subjects given that cognitive processes are regulated by executive functions [36]. Even though hubs were similar between groups, the development of cognitive networks in the children with epilepsy did not show the organization present in healthy subjects. These similarities in the number and nature of hubs along with altered developmental organization of the cognitive landmarks in the group of patients could represent why subjects with epilepsy improve with time in their cognitive testing but not as much as control subjects (Fig 1).

The differences in the prospective community networks might be viewed as implying that a degree of “cognitive reorganization” has taken place—a process that may be either adaptive or maladaptive. The fact that this network reorganization occurred in the context of what appears to be stable group differences with normal prospective developmental trajectories as defined by traditional neuropsychological analyses (Fig 1) implies that the reorganization is adaptive, serving to maintain cognitive development. While the pattern of prospective network change is clearly different between the epilepsy and control participants, it seemed able to maintain cognitive development at an age appropriate rate, at least for the first two years of the disorder.

Lastly, the integration between the subtests in the intelligence domain seems to be crucial for the optimal organization of cognitive development. A considerably high correlation between these four tests was observed in the group of controls that was absent in the group of patients. Also, a measure of executive function (D-KEFS Category Switching) presented a high integration to those tests of intelligence only in the group of controls. As has been demonstrated previously, epilepsy appears to disrupt a number of robust relationships observed in controls (e.g., birth weight and cognition [37], maternal IQ and child IQ [38]) that are absent in children with epilepsy.

Normal cognitive development requires an optimal balance between specialization of certain cognitive abilities (i.e., segregation) and amalgamation of multiple cognitive domains to improve cognitive efficiency (i.e., integration). The lack of balance between segregation and integration in the group of children with epilepsy as observed here could have important clinical implications. For example, lower segregation in their cognitive networks could indicate that improvement in a given test of a certain domain might not necessarily mean an improvement in another (related) test of the same domain as might be observed in normally developing children, but they improve separately. Also, low integration reinforces the lack of/reduced interdependence between different tests across different domains. Such reduced integration and segregation might even be an adaptive process that prevents deficiencies in a certain test to influence another test result. Our findings imply that future cognitive intervention should target specific abilities (e.g. response inhibition) rather than attempting to broadly improve cognitive domains (e.g. executive function).

An important point to notice is the heterogeneity of the epilepsy group, not only regarding the syndrome (ILRE or IGE) but also regarding the laterality of epilepsy foci in the group with ILRE. It is well documented in the literature that location of focal discharges could influence different cognitive abilities concerning the affected location (for a review, see [39]). Such distinction was not provided in this investigation, however is important that the reader interpret the results taking into consideration such heterogeneity.

In summary, the children with epilepsy deviated markedly from controls in terms of prospective developmental changes in the degree of their cognitive integration and segregation, while presenting the same number and similar nature of hubs. The combination of lower integration and segregation along with local similarities (hubs) in the development of cognitive landmarks observed in children with epilepsy when compared to controls could be contributing to the lower cognitive proficiency observed in pediatric epilepsies, but serving to maintain parallel improvement in the children with epilepsy over time.

## Conclusions

As we have shown previously, when examining individual test scores over the same time period, and longer (out to 5 years), a pattern characterized by static control vs. epilepsy test score differences are observed and maintained over time—neither worsening nor improving. But the metrics reported here demonstrate that there are very significant changes in the developmental inter-relationship between test metrics/abilities and the general connectedness and sophistication of the maturation of cognitive networks—a very different and dynamic picture of cognitive circuitry, all of which appears to be altered in children with epilepsy.

Thus, presentation and comparison of standard test metrics does not convey the dynamic and changing complexity in the interrelationships among discrete cognitive skills, the maturation of the cognitive network over time, and the differences between groups. From a neuropsychological perspective, the children with epilepsy are “maintaining” their overall cognitive skills, but in a very different fashion compared to controls. These changing dynamics in cognitive networks over time surely has a neurobiological contribution and a future challenge is to relate developments in the cognitive network to alterations in brain networks and lack thereof.

The next step in this line of research will be to interrogate the impact of epilepsy syndrome, and laterality of focal epilepsy syndromes, on baseline and prospective cognitive networks as characterized by the analytic methods presented here. Both the IGE and LRE groups are composed of several distinct syndromes and the similarities and differences across these syndromes will be an important line of inquiry going forward. Furthermore, the children with epilepsy examined here can be best characterized as what has been referred to as “epilepsy only”. How these results may differ compared to, for example, children with more intractable seizures and/or children with intellectual disability, or other clinically important subgroups, remain to be characterized.

## References

[1] Jackson DC, Dabbs K, Walker NM, Jones JE, Hsu DA, Stafstrom CE, et al The neuropsychological and academic substrate of new/recent-onset epilepsies. J Pediatr. 2013;162(5):1047–1053.e1. 10.1016/j.jpeds.2012.10.046 23219245 PMC3615134

[2] Fastenau PS, Johnson CS, Perkins SM, Byars AW, deGrauw TJ, Austin JK, et al Neuropsychological status at seizure onset in children: risk factors for early cognitive deficits. Neurology. 2009;73(7):526–534. 10.1212/WNL.0b013e3181b23551 19675309 PMC2730794

[3] Oostrom KJ, Smeets-Schouten A, Kruitwagen CL, Peters AC, Jennekens-Schinkel A; Dutch Study Group of Epilepsy in Childhood. Not only a matter of epilepsy: early problems of cognition and behavior in children with "epilepsyonly"—a prospective, longitudinal, controlled study starting at diagnosis. Pediatrics. 2003;112(6 Pt 1):1338–1344. 14654607 10.1542/peds.112.6.1338

[4] Oostrom KJ, van Teeseling H, Smeets-Schouten A, Peters AC, Jennekens-Schinkel A; Dutch Study of Epilepsy in Childhood (DuSECh). Three to four years after diagnosis: cognition and behaviour in children with 'epilepsy only'. A prospective, controlled study. Brain. 2005;128(Pt 7):1546–1555. 15817514 10.1093/brain/awh494

[5] Rathouz PJ, Zhao Q, Jones JE, Jackson DC, Hsu DA, Stafstrom CE, et al Cognitive development in children with new onset epilepsy. Dev Med Child Neurol. 2014;56(7):635–641. 10.1111/dmcn.12432 24650092 PMC4057956

[6] Craik FI, Bialystok E. Cognition through the lifespan: mechanisms of change. Trends Cogn Sci. 2006;10(3):131–138. 16460992 10.1016/j.tics.2006.01.007

[7] Bullmore E, Sporns O. The economy of brain network organization. Nat Rev Neurosci. 2012;13(5):336–349. 10.1038/nrn3214 22498897

[8] Rubinov M, Sporns O. Complex network measures of brain connectivity: uses and interpretations. Neuroimage. 2010;52(3):1059–1069. 10.1016/j.neuroimage.2009.10.003 19819337

[9] Bassett DS, Bullmore ET. Human brain networks in health and disease. Curr Opin Neurol. 2009;22(4):340–347. 10.1097/WCO.0b013e32832d93dd 19494774 PMC2902726

[10] Jaeggi SM, Buschkuehl M, Jonides J, Shah P. Short- and long-term benefits of cognitive training. Proc Natl Acad Sci U S A. 2011;108(25):10081–10086. 10.1073/pnas.1103228108 21670271 PMC3121868

[11] Goldin AP, Hermida MJ, Shalom DE, Elias Costa M, Lopez-Rosenfeld M, Segretin MS, et al Far transfer to language and math of a short software-based gaming intervention. Proc Natl Acad Sci U S A. 2014;111(17):6443–6448. 10.1073/pnas.1320217111 24711403 PMC4035955

[12] Wilson RS, Segawa E, Hizel LP, Boyle PA, Bennett DA. Terminal dedifferentiation of cognitive abilities. Neurology. 2012;78(15):1116–1122. 10.1212/WNL.0b013e31824f7ff2 22491858 PMC3320052

[13] Bonilha L, Nesland T, Martz GU, Joseph JE, Spampinato MV, Edwards JC, et al Medial temporal lobe epilepsy is associated with neuronal fibre loss and paradoxical increase in structural connectivity of limbic structures. J Neurol Neurosurg Psychiatry. 2012;83(9):903–909. 10.1136/jnnp-2012-302476 22764263 PMC3415309

[14] Vaessen MJ, Braakman HM, Heerink JS, Jansen JF, Debeij-van Hall MH, Hofman PA, et al Abnormal modular organization of functional networks in cognitively impaired children with frontal lobe epilepsy. Cereb Cortex. 2013;23(8):1997–2006. 10.1093/cercor/bhs186 22772649

[15] Bernhardt BC, Chen Z, He Y, Evans AC, Bernasconi N. Graph-theoretical analysis reveals disrupted small-world organization of cortical thickness correlation networks in temporal lobe epilepsy. Cereb Cortex. 2011;21(9):2147–2157. 10.1093/cercor/bhq291 21330467

[16] Ibrahim GM, Morgan BR, Lee W, Smith ML, Donner EJ, Wang F, et al Impaired development of intrinsic connectivity networks in children with medically intractable localization-related epilepsy. Hum Brain Mapp. 2014;35(11):5686–5700. 10.1002/hbm.22580 24976288 PMC6869397

[17] Kellermann TS, Bonilha L, Lin JJ, Hermann BP. Mapping the landscape of cognitive development in children with epilepsy. Cortex 2015;66:1–8. 10.1016/j.cortex.2015.02.001 25776901 PMC4405468

[18] Hermann B, Jones J, Sheth R, Dow C, Koehn M, Seidenberg M. Children with new-onset epilepsy: neuropsychological status and brain structure. Brain. 2006;129(Pt 10):2609–2619. 16928696 10.1093/brain/awl196

[19] Boersma M, Smit DJ, Boomsma DI, De Geus EJ, Delemarre-van de Waal HA, Stam CJ. Growing trees in child brains: graph theoretical analysis of electroencephalography-derived minimum spanning tree in 5- and 7-year-old children reflects brain maturation. Brain Connect. 2013;3(1):50–60. 10.1089/brain.2012.0106 23106635

[20] Song J, Nair VA, Gaggl W, Prabhakaran V. Disrupted Brain Functional Organization in Epilepsy Revealed by Graph Theory Analysis. Brain Connect. 2015 6;5(5):276–83. 10.1089/brain.2014.0308 25647011 PMC4490776

[21] Wang J, Zuo X, He Y. Graph-based network analysis of resting-state functional MRI. Front Syst Neurosci. 2010;4:16 10.3389/fnsys.2010.00016 20589099 PMC2893007

[22] Newman MEJ. The structure and function of complex networks. SIAM Review 2003;45,167–256.

[23] Humphries MD, Gurney K. Network ‘Small-World-Ness’: A Quantitative Method for Determining Canonical Network Equivalence. PLoS ONE 2008;3(4):e2051. 10.1371/journal.pone.0002051 PMC2323569 18446219

[24] Blondel VD, Guillaume J-L, Lambiotte R, Lefebvre E. Fast unfolding of communities in large networks. J. Stat. Mech 2008, P10008.

[25] Boccaletti S, Latora V, Moreno Y, Chavez M, Hwang D.-U. Complex networks: Structure and dynamics. Phys Rep, 2006;424(4–5);175–308.

[26] Sporns O, Honey CJ, Kötter R. Identification and classification of hubs in brain networks. PLoS One 2007;2(10). 10.1371/journal.pone.0001049 PMC2013941 17940613

[27] Wechsler D. Wechsler abbreviated scale of intelligence San Antonio, TX: The Psychological Corporation; 1999.

[28] Delis DC, Kaplan E, Kramer JH. Delis–Kaplan executive function system San Antonio, TX: The Psychological Corporation; 2001.

[29] Williams KT. Expressive Vocabulary test Circle Pines, MN: American Guidance Service; 1997.

[30] Wechsler D. Wechsler Intelligence Scale for Children. 3rd ed. San Antonio, TX: The Psychological Corporation; 1991.

[31] Lin JJ, Dabbs K, Riley JD, Jones JE, Jackson DC, Hsu DA, et al Neurodevelopment in new-onset juvenile myoclonic epilepsy over the first 2 years. Ann Neurol. 2014;76(5):660–668. 10.1002/ana.24240 25087843 PMC4362677

[32] Zhao Q, Rathouz PJ, Jones JE, Jackson DC, Hsu DA, Stafstrom CE, et al Longitudinal trajectories of behavior problems and social competence in children with new onset epilepsy. Dev Med Child Neurol. 2015;57(1):37–44. 10.1111/dmcn.12549 25040537 PMC4268257

[33] Fair DA, Cohen AL, Power JD, Dosenbach NU, Church JA, Miezin FM, et al Functional brain networks develop from a "local to distributed" organization. PLoS Comput Biol. 2009;5(5):e1000381 10.1371/journal.pcbi.1000381 19412534 PMC2671306

[34] Hagmann P, Sporns O, Madan N, Cammoun L, Pienaar R, Wedeen VJ, et al White matter maturation reshapes structural connectivity in the late developing human brain. Proc Natl Acad Sci U S A. 2010;107(44):19067–19072. 10.1073/pnas.1009073107 20956328 PMC2973853

[35] Lim S, Han CE, Uhlhaas PJ, Kaiser M. Preferential Detachment During Human Brain Development: Age- and Sex-Specific Structural Connectivity in Diffusion Tensor Imaging (DTI) Data. Cereb Cortex. 2015;25(6):1477–1489. 10.1093/cercor/bht333 24343892 PMC4428296

[36] Diamond A. Executive functions. Annu Rev Psychol. 2013;64:135–168. 10.1146/annurev-psych-113011-143750 23020641 PMC4084861

[37] Jackson DC, Lin JJ, Chambers KL, Kessler-Jones A, Jones JE, Hsu DA, et al Birth weight and cognition in children with epilepsy. Epilepsia. 2014;55(6):901–908. 10.1111/epi.12622 24735169 PMC4057970

[38] Walker NM, Jackson DC, Dabbs K, Jones JE, Hsu DA, Stafstrom CE, et al Is lower IQ in children with epilepsy due to lower parental IQ? A controlled comparison study. Dev Med Child Neurol. 2013;55(3):278–282. 10.1111/dmcn.12040 23216381 PMC3570624

[39] Fastenau PS. Transient cognitive impairment: impact of interictal epileptiform discharges on neuropsychological functioning and implications for clinical care and research In Helmsteadter C, Hermann BP, Lassonde M, Kahane P, Arzimanoglou A (Eds). Neuropsychology in the care of people with epilepsy (pps 69–92). John Libyy Eurotext, Montrouge, France, 2011.

